# Consciousness and Energy Processing in Neural Systems

**DOI:** 10.3390/brainsci14111112

**Published:** 2024-11-01

**Authors:** Robert Pepperell

**Affiliations:** Fovolab, Cardiff Metropolitan University, Cardiff CF5 2YB, UK; rpepperell@cardiffmet.ac.uk

**Keywords:** consciousness, energy, matter, brain, physics, neuroscience, complexity, biophysics, quantum mechanics, classical mechanics

## Abstract

Background: Our understanding of the relationship between neural activity and psychological states has advanced greatly in recent decades. But we are still unable to explain conscious experience in terms of physical processes occurring in our brains. Methods: This paper introduces a conceptual framework that may contribute to an explanation. All physical processes entail the transfer, transduction, and transformation of energy between portions of matter as work is performed in material systems. If the production of consciousness in nervous systems is a physical process, then it must entail the same. Here the nervous system, and the brain in particular, is considered as a material system that transfers, transduces, and transforms energy as it performs biophysical work. Conclusions: Evidence from neuroscience suggests that conscious experience is produced in the organic matter of nervous systems when they perform biophysical work at classical and quantum scales with a certain level of dynamic complexity or organization. An empirically grounded, falsifiable, and testable hypothesis is offered to explain how energy processing in nervous systems may produce conscious experience at a fundamental physical level.

## 1. Introduction

“All that we know about matter relates to the series of phenomena in which energy is transferred from one portion of matter to another, till in some part of the series our bodies are affected, and we become conscious of a sensation”.

This statement from James Clerk Maxwell, written in the second half of the nineteenth century, still presents a fundamental challenge for science [1]. If conscious experience is a physical process, and physical processes entail the transfer of energy from one portion of matter to another in material systems, then how does energy transfer—or energy processing more generally—in the organic matter of the brain produce conscious experience in us?

Enormous progress has been made in the scientific study of the brain and consciousness in recent decades, and there has been a proliferation of theories that seek to explain how neural processes can produce conscious experiences; for recent reviews see [2,3,4,5]. Several of these theories, such as Integrated Information Theory (or IIT), Global Workspace Theory (or GWT), the Orchestrated Objective Reduction Theory (or Orch OR), and Predictive Processing theory (or PP), have amassed support from empirical studies and attracted widespread theoretical interest [6,7,8].

Yet despite the empirical evidence, mathematical models and theoretical arguments that can be marshalled in favor of these approaches, questions have been raised about whether any of these theories, or any competing theory, can explain how certain patterns of neural activity produce conscious experiences [4,9,10,11,12,13]; for a critical comparison of several leading theories see [14]. Moreover, the comparative analysis of several leading biological theories has shown that each makes different predictions about the location of the brain activity associated with conscious processing [15]. All this suggests that we are a long way from agreement about the physical basis of consciousness and still lack a testable theory that is consistent with the established principles of physics, chemistry, and biology. In fact, it seems that the number and diversity of competing theories in the consciousness research “landscape” is proliferating rather than consolidating [5].

To address this problem further, this paper proposes that consciousness can be conceived in terms of physical processes that depend on interactions between energy and matter. As Maxwell states above, it seems to be a basic fact of nature that all physical processes at all scales entail energy transfer, transduction, and transformation between portions of matter. The same must also apply to the production of consciousness in nervous systems—assuming it is fundamentally a physical process. An explanatory framework for consciousness grounded in this fact would have two main advantages: first, it would be compatible with our theoretical understanding of all other physical processes in physics, chemistry, and biology and, second, it would help to unify the highly fragmented landscape of current consciousness research since all current approaches, in one way or another, would be expressions of this same basic fact.

The paper outlines its proposal in three main sections. First, several examples are provided that illustrate how nervous systems and brains function at multiple spatiotemporal scales—from whole brain to molecular to quantum level—as physical systems that transfer, transduce and transform energy between portions of organic matter to perform biophysical work that serves the interests of the conscious agent; conscious visual perception is one case. Second, recent neurobiological evidence is considered, which shows that a specific dynamic organization of this biophysical work is necessary to produce conscious experiences in the nervous system of sentient agents. Third, drawing on this evidence, a hypothesis is presented that may help to explain the relationship between energy processing activity in nervous systems and the onset of consciousness. A brief discussion follows on how the hypothesis can be tested and falsified.

## 2. Energy Processing in Neural Systems

The nervous system, including the brain, can be considered as a material system in the sense defined by Maxwell that is composed of organic matter and which utilizes electrochemical energy in a highly efficient way [1,16]. During neural activity, the nervous system transfers, transduces, and transforms energy between portions of its matter at multiple scales to perform bio-chemo-physical work (biophysical work for short) that serves the behavioral needs of the organism, including the production of its sensations and conscious experiences. This section briefly considers some examples of this energy transfer at different scales, from the level of the nervous system globally to quantum level processes occurring within sub-cellular structures.

### 2.1. Energy Processing in Nervous Tissue

Sensory experience normally begins with environmental stimulation. Taking visual perception as a paradigmatic example, visually equipped animals are immersed in what the perceptual psychologist James J. Gibson [17] called an “sea of physical energy”. In the case of vision, this consists of certain visible frequencies of electromagnetic light energy that permeate the environment, and which can be used by the organism to sense external conditions and act accordingly. Sensory receptors in all modalities across the body work by transducing various forms of environmental energy gradient—electromagnetic, thermal, mechanical, gravitational, and chemical—into electrochemical neural activity. In the case of the eyes, it is the reaction of light sensitive opsin receptors in the retinas to the arrival of photons that transduces electromagnetic energy from the environment into electrochemical energy in the eye [18]. This stimulation triggers a cascade of neural activity that consists of the further transfer of electrical, chemical, photonic, and mechanical energy within and between cells in the animal’s visual system. The biophysical work thus performed reconfigures the organic matter of the system at various scales to actuate the relevant adaptive functions [19,20].

The central nervous systems of animals, and especially their brains, are astronomically complex in structure and behavior; the unique complexity of the human brain, which has organizational scales spanning at least 10 orders of magnitude [21,22], presents a particularly formidable challenge for those seeking to understand its functions and products [23,24]. But nervous systems and brains, no matter how complex and multiscale, are like other material systems in that they consist of portions of matter that are acted on by forces when energy is transferred, transduced, and transformed during the performance of work.

In addition to the water that makes up around 75% of the chemical composition of the adult brain and spinal cord [25], human nervous tissue consists largely of fats and proteins [26,27], while the fundamental force that actuates most of its biophysical work is electromagnetic due to chemical gradients, electrical currents, and endogenous electromagnetic fields. Gravitational forces can also play a role [28,29].

As with the rest of the body, the primary source of energy for the nervous system is glucose delivered through its blood vessels and converted to free energy by the hydrolysis of adenosine triphosphate (ATP) molecules [30]. The nervous system has a vast repertoire of processes that metabolize this source of free energy to carry out biophysical work. Some of the most widely studied are the active transport of ions across neuronal membranes by sodium–potassium pumps, the generation of electrical currents along axons during action potentials, the transport of neurotransmitter molecules across synaptic clefts, and the propagation of extracellular fields [31,32,33].

### 2.2. Energy Processing in Brains

The fact that brains do a relatively large amount of the body’s biophysical work is evidenced by the fact that in humans the brain consumes some 20% of the entire energy budget of the body at rest, while accounting for only 2% of adult body mass [34]. This high rate of metabolic turnover in the brain is necessary to sustain an equally high rate of biophysical work—the majority of which is devoted to non-task related spontaneous activity [35]. The visual processing regions of the brains of many creatures are particularly energy consuming [36].

Much modern neuroscientific research, including that which studies the neural underpinnings of visual perception, depends on a range of non-invasive imaging techniques such as functional magnetic resonance imaging (fMRI), magnetoencephalography (MEG), and electroencephalography (EEG) to study in vivo brain organization and behavior, often in response to external stimulation [37]. These techniques work by detecting and measuring changes in energy consumption or distribution in the living brain, either directly or indirectly, to track neural activity and anatomical function. fMRI, for example, detects changes in blood flow associated with increases in neural activity, which is an indirect measure of neural energy metabolism [38]. EEG, meanwhile, directly measures changes in electromagnetic field energy due to fluctuations in electrical potentials across the cortex [39].

Neural activity that correlates with sensory stimulation is widely thought to be responsible for the behaviors or functions associated with the stimulus response. For example, the fact that certain regions in the cerebral cortex are activated when a person is visually stimulated is taken as evidence that this increase in neural activity is, at least in part, responsible for the person’s subjective experience of the stimulus [37]. Given how imaging techniques operate, the neuroimaging of stimulus evoked neural activity is in fact observing, directly or indirectly, the biophysical work that correlates with the subjective phenomenon in question [35,40]. These examples further illustrate how the brain functions as an energy processing system that processes high levels of energy between portions of its matter when performing biophysical work.

It is important to note the stochastic nature of neural processing and its role in functions such as conscious vs. unconscious decision-making [41]. The tendency of a neuron or population of neurons to fire or not fire, and so lead to one course of action or another, has been shown to be probabilistic, being based on the dynamic integration of multiple different signals such that the neural processing system can occupy several states of stability. Depending on which state of stability the system is in, i.e., whether it is spontaneous, stable, or multi-stable, will affect its tendency to ‘jump’ over barriers in the system’s energy landscape, so influencing the evolution of the system.

### 2.3. Energy Processing in Neurons

There are an estimated 86 billion neurons in an adult human brain and a similar number of other kinds of cell [42], all of which exhibit a great deal of morphological and functional diversity [43]. Assuming these neurons comprise the elementary functional units of the brain, as per the ‘neuron doctrine’ [44], then we begin to grasp how multitudinous and diverse the processing of energy is at the individual neuronal level.

Neurons typically have a voltage potential of around −70 millivolts between the intra- and extracellular fluids separated by the cell membrane [45]. To regulate this potential, work is carried out on the ions that make up much of the cell’s matter by ATP-driven pumps embedded in the membrane and by the influx of ions through channels in the cell’s dendrites due to the action of neighboring cells. Moving ionic matter against chemical gradients and changing the conformation of ion channels consumes free energy and the work of regulating voltage potentials across the cell membrane and synaptic transmission accounts for much of the brain’s energy budget [46,47].

If the difference in charge across the membrane in a neuron reaches a critical threshold, then an action potential is triggered that conveys an electrical pulse along the cell’s axon, transferring energy to neighboring cells via synaptic junctions [48]. This excitatory process also consumes significant amounts of metabolic energy [31,49], although relatively little attention has been paid to evaluating the metabolic costs of interneuron inhibition work [50].

More recently discovered energy-driven mechanisms in the nervous system include the many kinds of nanoscale molecular motor that transport tiny cargos along subcellular structures [51], the mechanical forces that orchestrate brain development [52], the ephaptic coupling of cells at micro- and mesoscopic scales due to externally imposed and endogenous electromagnetic field fluctuations [53,54,55], and the long-range coupling between cells using membrane nanotubes to propagate electrical signals [56,57]. Ucar [58] reported muscle-like mechanical processes in dendritic spines that produce an estimated 10 nanonewtons of force and which may play a role in working memory. Such processes often entail the transformation of energy from its potential form—as stored in chemical bonds—to its kinetic form—as expressed in the motion of mechanical work.

**Figure 1:**
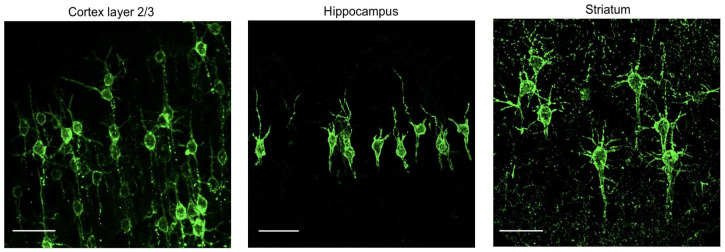
Biophysical work in neurons. These sample images from Piatkevich et al., 2019 [59] show groups of active neurons from three different brain regions illuminated by a voltage imaging technique.

A vivid illustration of the biophysical work being performed by individual neurons is provided by Piatkevich and colleagues [59] as shown in Figure 1. Using a modified molecule inserted into the membrane that emits light when the cell is electrically active, the team were able to image neural activity at high spatiotemporal resolutions.

### 2.4. Quantum Scale Energy Processing in Neural Structures

In a 1932 lecture entitled ‘Light and Life’, where he defined light as “transmission of energy between material bodies at a distance”, Neils Bohr proposed that quantum phenomena—which operate at nanometer and sub-nanometer scales—might play a functional role in living processes such as photosynthesis and vision [60]. Unlike in classical mechanics, much measurement of energy transfer in atomic, photonic, protonic, ionic, and molecular systems is quantized and probabilistic. But despite this fundamental difference, classical and quantum processes both entail the transfer, transduction, and transformation of energy to bring about changes in the configuration of matter to perform work [61].

Following Bohr’s speculations, interest in the functional role of quantum processes in biology, including neurobiology, has gradually intensified [62,63]. While controversies and uncertainties remain, evidence is accumulating that many biological processes are optimized to exploit quantum scale phenomena such as coherence, tunnelling, and surface hopping to efficiently harvest and process energy and usefully reconfigure matter as, for example, when separating transmembrane charges during photosynthesis [64].

In the field of neurobiology, John Eccles and Friedrich Beck made a proposal in the 1990s about the probabilistic role of quantum tunnelling in exocytosis in the production of consciously intended action through summation of vast quantities of microscopic energy transfers in synapses to influence mental events at the macro scale [65]. Following this early work, a field of quantum neurobiology has emerged which studies a wide range of neural phenomena at the nano- and sub-nanometer scales [21,66] and which has stimulated new approaches to longstanding neuroscientific problems, including understanding brain dynamics, cognition, and the mechanisms underlying consciousness [67,68,69].

One neural component that has attracted theoretical and experimental attention in this context is the microtubule, a nano-scale polymer packed in vast numbers into the neuron that performs several key roles, including the maintenance and modification of cell structure through exertion of forces, mitosis, and the transportation of macromolecules within the cell [22,70,71]. Recent experimental work has shown that microtubules are surprising effective light harvesters that are able to transfer energy over far greater distances than previously predicted [72]. Whilst it is not yet known if this capacity for long-range energy transfer is exploited by neurons for the performance of useful work, such as enabling cognition or consciousness, it is interesting to note that this effect is dampened by the presence of anesthetic agents, such as etomidate and isoflurane [72].

### 2.5. Neural Activity as Biophysical Work

To summarize, it has long been known that neural activity entails metabolically driven work that is intimately related to mental function and behavior [73,74]. During the performance of this work, matter is dynamically reconfigured at spatial multiple scales, both classical and quantum, in ways that serve the biological needs of the organism [75]. A few illustrative examples have been outlined here. Given the great variety and high rates of biophysical work performed by the components of the human brain in proportion to its contribution to the body’s total mass, this organ is the most densely saturated with biophysical work, particularly in the visual areas. Given also that energy processing activity in the brain is known to be causally associated with the production of consciousness it is reasonable to assume that this biophysical work is causally related to the emergence of conscious forms of experience.

## 3. Conscious Experience in the Brain

It has been argued here that the nervous system, and the brain, are prolific producers of biophysical work, which is generated by electrical and chemical activity occurring at multiple scales, from quantum to whole brain. How might the performance of this biophysical work lead to the production of conscious behaviors, such as visual experiences in the nervous system?

Healthy brains have relatively high metabolic rates and therefore perform profuse quantities of biophysical work even when we are not conscious, as in non-dreaming sleep or during anesthesia [76,77,78,79]. A global cerebral metabolic rate of at least 42% of normal cortical activity has been shown to be the minimum required to sustain consciousness [80]. But the brains of people who lose consciousness during epileptic seizures and following severe brain trauma still can have relatively high levels of metabolic activity [80,81]. In some cases, cortical activity rates even rise during loss of consciousness [82,83]. The performance of biophysical work in the brain, even at high levels, is therefore seemingly necessary not sufficient to produce consciousness. What then is the difference between a metabolically active brain that is performing biophysical work within the organic matter of its neurons but whose owner is unconscious and a brain whose owner is consciously experiencing? To address this question first requires a working definition of conscious experience.

### 3.1. Defining Conscious Experience

Defining consciousness for scientific or philosophical purposes is notoriously problematic, even though it is arguably the condition with which we are most intimately familiar in our waking and dreaming lives. The definition given by the philosopher Thomas Nagel [84], which is that there is ‘something it is like’ for a being or a system to consciously experience from its intrinsic perspective, is now widely used as shorthand for the essentially qualitative nature of a being’s private conscious experience. On this definition, being in a conscious state entails awareness of the being’s self or environment which, in turn, entails certain feelings or sensations for the being that accompany that awareness. This definition, however, treats consciousness as an entirely private, first-person phenomenon that cannot be observed or measured from a third-person perspective, so limiting its scientific value as an operational definition.

In some clinical situations it is necessary, even vital, to try and determine a person’s state of consciousness from a third-person perspective. Clinicians diagnosing patients with acute brain injury, for example, routinely determine the presence or impairment of consciousness using tools like the Glasgow Coma Scale (GCS), which categorizes levels of consciousness based on the patient’s response to external stimulation, whether auditory or tactile [85]. According to this method, the more complex the behavioral response elicited by stimulation, the more conscious the patient. However, as noted below, conscious states have been determined in people who are behaviorally unresponsive, and so complexity of behavioral response cannot be the sole criterion. Recently developed brain imaging-based assessment methods, also discussed below, have found correlations between the complexity of elicited neural response and levels of consciousness, and these methods are now gaining traction in the clinical setting by providing third-person measures of conscious states that do not depend on the behavioral response of the patient [86,87].

While the deep philosophical questions pertaining to the nature of consciousness—many of which have been debated for centuries—cannot be resolved here, it is possible to provide a useful operational definition for the purposes of scientific study and to test the hypothesis outlined below. The present operational definition is that consciousness is *the intrinsic state of awareness of a system which manifests as a complex behavioral or neurobiological response to external stimulation.* This operational definition is chosen because it combines the key properties of Nagel’s widely accepted definition with those of the clinical tools discussed above and provides a measurable criterion against which to test the hypothesis outlined below.

### 3.2. The Neural Organization of Conscious Experience

With this provisional definition of consciousness in mind we can address the question just posed: what differentiates a system that is performing biophysical work non-consciously from one that is working to produce states of conscious experience such as visual perceptions?

We do not yet understand the relationship between brain processes and conscious states well enough to give a definitive answer to this question. But we can *begin* to answer it empirically by turning to the wealth of evidence generated by recent neuroscientific studies in which the organization and behavior of the brains of people with different states or levels of conscious experience have been measured and compared. The broad conclusion from this evidence is that the specific way that neural activity in a brain—that is, the biophysical work performed at different spatiotemporal scales—is organized and how it behaves dynamically determines the state and level of conscious experience of its owner.

One of the first studies to show that measuring the dynamic organization of neural activity, or biophysical work, at the classical scale could reliably discriminate different levels of consciousness was conducted by Casali and colleagues [88]. Neural responses to energy influxes from transcranial magnetic stimulation were measured using EEG imaging in the brains of healthy people and those diagnosed as having impaired brain function. Patterns of activity that were measured as being statistically more complex, using a metric called the Perturbational Complexity Index (PCI), were found to be more likely to support conscious states than those having less complexity by the same metric.

This method of discriminating levels of consciousness from imaging data was subsequently validated [89], while several other complexity-based methods of measurement have since been developed [90,91]. Bodart and colleagues [86] measured metabolic activity using both PET and EEG signal complexity using PCI in patients with impaired consciousness. They found relatively high levels of metabolism and of complexity in four patients who were behaviorally unresponsive. These findings suggested that these measurement techniques may help to diagnose people with minimally conscious states that would otherwise not be detected by tools such as GCS.

This success of this and similar work in diagnosing and discriminating levels of consciousness using information theoretical methods applied to imaging data has led to a growing consensus about the organization of the neural activity that produces conscious states, which builds on long standing proposals about the relationship between consciousness and complexity in the brain [92,93]. In a recent review paper, Sarasso et al. [94] summarized this emerging “consilience of evidence” thusly:

“Overall, a large body of work supports the notion that the presence of consciousness is invariably associated with high brain complexity, which, vice versa is found to be consistently decreased during physiological, pharmacological, or pathological-induced loss of consciousness”.

It has been argued by proponents of IIT that this conclusion is supportive of their theoretical approach [6].

Another body of research has focused on the neural activity that seems to be reliably evoked when a stimulus is consciously perceived compared to when the same stimulus is not. One phenomenon of particular interest in this context is the so-called ‘ignition’ of an abrupt, nonlinear propagation of activity that occurs in the higher cortical areas of the brain between 200–300 milliseconds following the onset of consciously perceived stimulation, which some have argued is a neural signature of perceptual awareness [95,96,97].

This ignition phenomenon has been associated with recurrent or re-entrant feedback circuits that occur widely throughout the brain [98,99]. It has been suggested that these feedback circuits allow neurons, or ensembles of neurons, to dynamically modulate neural responses to stimulation as well as integrate activity across distributed functional regions [100,101]. More specifically, recurrent or re-entrant loops may enable long term reverberatory activity within neural populations following ignition, which may act to amplify and sustain the activity associated with a consciously perceived stimulus [99]. One reason that amplified and sustained reverberant activity may be implicated in the production of conscious experience is that it enables the neural response to a particular stimulus to be accessed more widely by other non-conscious processes. This allows multiple disparate cortical processes to bind together into a unified cognitive state, a process that has been observed unfolding in the brains of people recovering from general anesthesia [99]. It has been argued that these findings are supportive of the GWT approach [7].

Meanwhile, evidence is emerging about the role of quantum scale processes in neural function and the production of conscious experience [102,103,104]. It is becoming clearer how such processes might actuate states of simultaneous differentiation and integration within neural structures and so increase the complexity of the biophysical work being performed. The Orch OR theory of Penrose and Hameroff [67], for example, is perhaps the most prominent and highly developed theory of this kind. It proposes that the orchestration (integration) of vast numbers of distinct (differentiated) wave function collapse events occurring within neural microtubules, each of which is a distinct ‘proto-conscious’ event, gives rise to the holistic sense of conscious experience that the brain produces.

From a philosophical perspective, Chalmers has proposed a “principle of organizational invariance” (POI), in which it is the specific organization of a physical system that enables it to sustain states of consciousness, and that experience is an emergent property of the causal interactions between its parts [105]. While he refers mainly to the role of information processing, his arguments could also apply to energy processing.

In sum, the organization and behavior of dynamic multiscale activity or biophysical work occurring in the brain—that is, its dynamical complexity from quantum actions to the whole brain—and the level of that activity over prolonged periods have been shown experimentally to correlate with levels and kinds of conscious states: higher levels of more complex, more diverse, more coordinated, and more anti-coordinated patterns of neural activity are predictive of more conscious experiential states [106]. Moreover, this general pattern is compatible with several leading theoretical approaches to consciousness, namely IIT, GWT, Orch OR, PP, and POI.

### 3.3. Differentiation and Integration

The evidence discussed so far points towards a general principle underlying the organization and behavior of the necessity quantities of neural activity that produce conscious experience: it depends on having a certain level of neural activity that is suitably *differentiated*—due to it consisting of many distinct and diverse parts or processes—and *integrated*—due to these parts or processes cooperating, interacting, or affecting each other across space and over sufficiently long periods of time [107].

This general principle has been invoked to explain and model a range of neurophysiological and behavioral phenomena, including coordination dynamics underlying cognition and motor control [108], pharmacologically altered states of consciousness [109], minimally altered states of consciousness [110], the regulation of neural information flow [111], attentional control [112], disorders of consciousness [113], the pathology of psychiatric and neurodegenerative conditions [114], and schizophrenia [115], as well as consciousness itself [7,116,117,118].

A recent review paper on this topic concludes that it is the kind and level of differentiation and integration of its metabolically driven neural activity—that is, its dynamic complexity—rather than its gross volume that determines a given brain’s capacity for consciousness [119]. This may explain why animals can have conscious experiences, providing they have sufficiently differentiated and integrated neural activity, even though their brains can be far smaller than those of humans [120,121], and why some people and animals with little or no cerebral cortex exhibit complex behavior and feelings [122,123]. More recently, it has been shown that measuring the balance between integration and differentiation (or segregation) can reliably discriminate conscious from non-conscious states in humans [87].

Measurements of the dynamical complexity of neural activity—specified in terms of the kind, level, and balance of differentiation and integration across space and over time—and the associated complexity of behavioral responses to stimulation, do not in themselves explain why certain patterns of brain organization produce conscious experiences. But they point to a potentially important principle that can be used to extend our current explanatory arsenal.

## 4. The Production of Conscious Experience in Neural Systems

To begin to explain the production of consciousness as a physical process occurring in the nervous system, it is necessary to formulate a testable hypothesis. In this section, a hypothesis is presented, and consideration briefly given to how it might be tested.

### 4.1. Hypothesis

The neurobiological evidence cited here, particularly that relating to energy processing in the organic matter of nervous systems, suggests the following hypothesis that may help to explain the biological nature of consciousness: *States of consciousness are produced by the biophysical work performed within nervous systems and depend on how that biophysical work is dynamically organized, as measured by the differentiation and integration of the neural energy processing activity.*

This hypothesis has explanatory value because it may help us to account for the production of consciousness in organic beings with the same physical principles that are used elsewhere in science to account for non-organic and non-conscious phenomena, namely the principles that govern the transfer, transduction, and transformation of energy in material systems. It may also help to explain what differentiates a nervous system that is performing biophysical work non-consciously from one that is doing so consciously and even what differentiates a system undergoing one kind of conscious experience from another. To illustrate this, consider the following ways in which the dynamic organization of a nervous system might affect the level of consciousness produced by that system:

1. When biophysical work is performed in localized portions, or subsystems, of a nervous system without coordination between the subsystems, then the system is differentiated or segregated but not spatially or temporally integrated. Since the biophysical work being performed in the nervous system is insufficiently differentiated and integrated, the system is unconscious: there is nothing ‘it is like’ as the system as a whole and the stimulation of the system elicits a simple linear response.
2. When different subsystems at different scales interact by transferring energy between themselves over longer periods in coordinated ways, they become integrated into larger and more diverse subsystems at different spatiotemporal scales. The nature of this larger subsystem is determined by the way it integrates the biophysical work of its subsystems over time. But below a certain critical threshold of differentiation and integration of its activity, it remains unconscious: there is ‘nothing it is like’ as the system and the stimulation of the system elicits a less simple but still linear response.
3. Once the nervous system has reached or exceeded a critical level of integration and differentiation of its biophysical work then there is ‘something it is like’ as the system from its intrinsic perspective, and it reaches the threshold necessary to be a consciously experiencing system. The stimulation of the system then elicits a complex nonlinear response, whether neurobiologically or behaviorally. The quantity and complexity of the work being carried out in each system, including all its subsystems, determines the level or kind of conscious experience produced in the system as a whole and the complexity of its response to stimulation.

**Figure 2:**
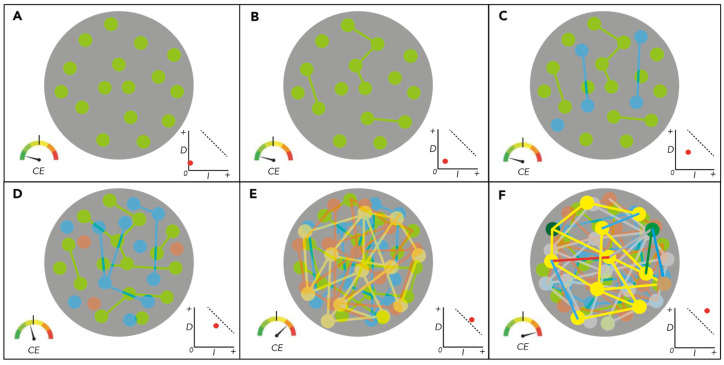
Illustration of a nervous system evolving from non-conscious to conscious states. In this highly simplified scale free model, panel (**A**) shows a material system, such as a nervous system, indicated by the grey circle that consists of several differentiated subsystems in which biophysical work is being performed, indicated by the green dots. As this energy processing is not dynamically integrated, the system is far below the threshold sufficient for conscious experience, indicated by the black bar on the meter labelled CE in the lower left of the panel. The graph in the bottom right of the panel plots as a red dot the relationship between the amount of differentiation (vertical axis) and integration (horizontal axis) in the system, and the threshold of sufficient differentiation and integration for conscious experience is indicated by the dashed slope. In panels (**B**–**D**), the level of activity and the amount of differentiation and integration are indicated by the lines, and the system gradually increases as different layers of subsystems, indicated with different colors, work, and interactions. But the threshold for conscious experience is not reached. In panel (**E**), the levels of activity and amounts of dynamic differentiation and integration in the system produce conscious experience, as indicated by the meter and the graph. In panel (**F**), a different pattern of subsystem activity is integrated in a different way from panel (**E**), which represents a different conscious experience for the system overall. Moreover, the increased vividity of the colors that represent the subsystems and their interactions in panel (**F**) and the more highly integrated and differentiated interactions between different layers of the subsystems indicate that greater quantities of more complex work are being carried out; the conscious experience is therefore more intense and richer than that in panel (**E**).

The main principle of system organization in the hypothesis is illustrated in a simplified model in Figure 2.

### 4.2. Testing and Falsifying the Hypothesis

The hypothesis introduced here focuses on the role of biophysical work in the production of consciousness in nervous systems at multiple spatiotemporal scales. More specifically, it suggests that it is the dynamic organization of that biophysical work, as measured by its levels of integration and differentiation, that distinguishes non-conscious from conscious states of the system. In order to test this hypothesis, therefore, it is necessary to focus on the measurements of biophysical work and energy processing activity at multiple scales and on the levels of consciousness—as determined by the complexity of response to external stimulation—that correspond to different measures.

Recent advances in neuroscientific techniques and methods offer many ways to measure and model the production of conscious experience in living systems and test the hypothesis. Practically, this would require apparatus sensitive enough to analyze and control energy processing and biophysical work within and between neural structures at quantum and classical scales to observe and manipulate the electrical potentials, chemical gradients, electromagnetic fields, and quantum-scale energy transfers, through which forces are exerted on the system’s organic matter. Mapping the specific patterns of biophysical work in organic matter to specific experiential states as expressed by specific outward behavior or subjective reports, and then controlling these patterns to predictably produce new experiential states, would provide a method of answering specific research questions.

Such experiments could be conducted by manipulating levels or patterns of energy transfer, transduction, and transformation in nervous systems using targeted brain stimulation [124,125] or optogenetic control [59,126] and measuring the resulting neural activity using a range of imaging and recording techniques, such as electrocorticography [127] or electroencephalography [128], or by calculating quantities of energy transfer within and between neurons [16,34,129,130,131].

Methods and tools for measuring quantum effects in neurobiological systems in vivo are also developing, allowing the investigation of energy transfer and biophysical work at nanometer scales. For example, fluorescence lifetime microscopy and Förster resonance energy transfer enable researchers to study interactions between proteins and conformational changes in proteins when energy from light acts on quantum-scale matter [132]. The resulting data from experiments conducted using such tools could be modelling with a host of mathematical and statistical modelling methods, all allied to ever growing computational power such as graph theory, mutual information theory, and methods from statistical mechanics, for example [133,134,135,136].

The proposed hypothesis could be falsified in the following ways. First, if it can be shown that nervous system *A* is performing biophysical work of a certain organizational complexity, as measured by its kind, level, and balance of integration and differentiation, and is in a conscious state while nervous system *B* is performing biophysical work of equal dynamic complexity yet is not in a conscious state, then the hypothesis would be undermined. Second, if it can be shown that a system is not performing biophysical work, i.e., is not processing energy, yet is in a conscious state, then the hypothesis will be undermined. Proponents of IIT have argued that ‘silent’ or inactive systems of logic gates would be conscious if correctly arranged [6,137]. The present hypothesis suggests that conscious systems require the presence of energy-driven activity, so contradicting this claim of IIT.

## 5. Further Work

This paper does not aim to provide a comprehensive account of the general principles discussed. For reasons of space and to aid in the clarity of exposition, important issues have been dealt with only briefly or omitted. For instance, the hypothesis has implications for several other neurobiological theories of consciousness than those mentioned above, especially those such as electromagnetic field and resonance theories that emphasize the role of energy in the production of consciousness [138,139,140]. However, the fundamental nature of the explanatory principles outlined here does provide grounds for the potential unification of these and many other theories of consciousness.

To consider one example, the Predictive Processing framework, as developed by Karl Friston and colleagues, is evolving from what is primarily a theory of perception, cognition and action into one that aspires to explain sensation, sentience, and consciousness [141]. It rests heavily on the Free Energy Principle, which is an information theoretical measure of surprisal or uncertainty—i.e., the difference between the actual and the expected input to a system that is operating on Bayesian probabilistic principles and which is seeking to minimize this difference in order to optimize its internal model of its external environment—rather than metabolic energy processing, as discussed here [142].

However, in a more recent paper, Friston and colleagues have extended this framework in an approach called ‘Markovian monism’, which, they argue, may contribute to our understanding of the origins of consciousness [143]. They employ the concept of the Markov blanket, which is a mathematical way of describing the boundary or membrane that acts both as a barrier differentiating a living system from its environment and as a bidirectional information conduit that integrates it into its environment. The monism in this case refers to the claim that the internal states of the system, and any properties of sentience it may have, are made of the same thing as its environment, this being what they describe as ‘information geometry’. Mind and matter, in their view, are dual aspects—one intrinsic and one extrinsic—of this same information geometry.

While this is presented as a mathematical information-processing model, its principles might equally apply to the energy-processing framework outlined here, where energy transfer, transduction, and transformation perform the role as the monistic causal agent of perception and sentience. Although space does not allow a detailed discussion of the parallels between the two approaches, Markovian monism offers a mathematical framework within which to model the flow of energy in the environment, between the environment and the organism (across its Markov blanket) and within the organism, to the point where it is organized in such a way as to produce sentience or consciousness.

A major challenge that remains for the approach discussed in this paper is to understand how different patterns of biophysical work in different areas of the brain produce distinct kinds of conscious experiences; what, for example, makes a certain pattern of activity in a certain brain region feel like a visual experience rather than an auditory experience, or what makes a particular part of a visual experience appear red rather than green? Accounting for the enormous variety of modes and features of perceptual experience is daunting enough without considering the many other features of conscious experience such as memory, attention, cognition, emotion, and imagination that would also need to be accounted for in the same way.

A key future task is to establish appropriate technical protocols for measuring and modelling biophysical work in systems of varying complexity; the hypothesis can only prove its explanatory value if it can be tested experimentally and modelled mathematically. I have attempted to show that existing empirical evidence generated by different theoretical approaches to consciousness can be interpreted in a way that is consistent with this energy–based approach and that existing mathematical frameworks can be adapted to model energy processing in nervous systems. But this previous work has not been designed to test or model this hypothesis specifically, and doing so will require new experimental paradigms, and perhaps new research questions, or at least reframing existing questions.

The most important research question that needs to be reframed, I would suggest, is the one that currently asks “what are the neural correlates or signatures of conscious experience?” [4,116]. Many current neuroscientific explorations of this question are conducted on the assumption that the brain functions essentially as an information processing system in much the same way as a digital computer. Here I have tried to show that the brain can also be treated as an energy-processing system (the two conceptions are complementary rather than competing; see [144]) and, moreover, that such a treatment can yield explanations for those theories grounded in the computational paradigm.

The research question can then be rephrased as follows: “What patterns of energy processing between portions of organic matter in an animal’s nervous system produce conscious experience in that system?” or more generally (given the theoretical possibility of artificially conscious machines): “What patterns of energy transfer between portions of matter in a material system produce conscious experience in that system?” Any conclusive answer to this question would contribute significantly to our understanding of consciousness as a physical process.

## 6. Conclusions

This paper offers no definite conclusions on the question of how certain patterns of energy transfer in certain material systems produce certain consciously experienced states in those systems. Rather, its main purpose has been to address the fundamental challenge posed by the statement from James Clerk Maxwell quoted at the outset. It has achieved this by framing the question in terms of the energy transferred, transduced, and transformed when biophysical work is performed on portions of organic matter in nervous systems and which, in the case of vision, can lead to vivid and meaningful visual experiences.

Framing the challenge in this way and proposing experimental approaches that aim to investigate the hypothesis derived from it may help us to understand this most difficult of scientific problems, as I have argued, not least because it treats conscious experience—which is at once vividly present to us and yet explanatorily elusive—as a product of physical processes in the same way as the many other established classical- and quantum-scale phenomena described in natural science. Moreover, the fundamental nature of this approach offers an opportunity to establish common ground between competing and seemingly incompatible theories of consciousness.

## Data Availability

The original contributions presented in the study are included in the article, further inquiries can be directed to the author.

## References

[1] Maxwell J.C. (1877). Matter and Motion.

[2] Northoff G., Lamme V. (2020). Neural signs and mechanisms of consciousness: Is there a potential convergence of theories of consciousness in sight?. Neurosci. Biobehav. Rev..

[3] Signorelli C., Szczotka J., Prentner R. (2021). Explanatory profiles of models of consciousness—Towards a systematic classification. Neurosci. Conscious..

[4] Seth A.K., Bayne T. (2022). Theories of consciousness. Nat. Rev. Neurosci..

[5] Kuhn R.L. (2024). A landscape of consciousness: Toward a taxonomy of explanations and implications. Prog. Biophys. Mol. Biol..

[6] Tononi G., Boly M., Massimini M., Koch C. (2016). Integrated information theory: From consciousness to its physical substrate. Nat. Rev. Neurosci..

[7] Mashour G.A., Roelfsema P., Changeux J.P., Dehaene S. (2020). Conscious Processing and the Global Neuronal Workspace Hypothesis. Neuron.

[8] Sprevak M., Smith R. (2023). An Introduction to Predictive Processing Models of Perception and Decision-Making. Top. Cogn. Sci..

[9] Brown R. (2017). Integrated Information Theory Doesn’t Address the Hard Problem. https://onemorebrown.com/2017/08/13/integrated-information-theory-doesnt-address-the-hard-problem/.

[10] Cerullo M.A. (2015). The Problem with Phi: A Critique of Integrated Information Theory. PLoS Comput. Biol..

[11] Merker B., Williford K., Rudrauf D. (2021). The integrated information theory of consciousness: A case of mistaken identity. Behav. Brain Sci..

[12] Brown R., Lau H., LeDoux J.E. (2019). Understanding the Higher-Order Approach to Consciousness. Trends Cogn. Sci..

[13] Schurger A., Graziano M. (2022). Consciousness explained or described?. Neurosci. Conscious..

[14] Doerig A., Schurger A., Herzog M.H. (2021). Hard criteria for empirical theories of consciousness. Cogn. Neurosci..

[15] Yaron I., Melloni L., Pitts M., Mudrik L. (2022). The ConTraSt database for analysing and comparing empirical studies of consciousness theories. Nat. Hum. Behav..

[16] Niven J.E. (2016). Neuronal energy consumption: Biophysics, efficiency and evolution. Curr. Opin. Neurobiol..

[17] Gibson J.J. (1979). The Ecological Approach to Visual Perception.

[18] Pepperell R. (2002). Vision as an Energy-Driven Process. arXiv.

[19] Yoshioka T., Sakakibara M. (2013). Physical aspects of sensory transduction on seeing, hearing and smelling. Biophysics.

[20] Manicka S., Levin M. (2019). Modeling somatic computation with non-neural bioelectric networks. Sci. Rep..

[21] Swan M., dos Santos R.P., Witte F. (2022). Quantum Neurobiology. Quantum Rep..

[22] Hameroff S. (2021). ‘Orch OR’ is the most complete, and most easily falsifiable theory of consciousness. Cogn. Neurosci..

[23] Bassett D.S., Gazzaniga M.S. (2011). Understanding complexity in the human brain. Trends Cogn. Sci..

[24] Tognoli E., Kelso J.A. (2014). Enlarging the scope: Grasping brain complexity. Front. Syst. Neurosci..

[25] Forbes R.M., Cooper A.R., Mitchell H.H. (1953). The composition of the adult human body as determined by chemical analysis. J. Biol. Chem..

[26] Suzuki K., Suzuki Y. (1972). The Metabolic Basis of Inherited Disease.

[27] Gonzalez-Riano C., Garcia A., Barbas C. (2016). Metabolomics studies in brain tissue: A review. J. Pharm. Biomed. Anal..

[28] Kohn F.P., Koch C., Ritzmann R., Russomano T., Rehnberg L. (2018). The Effect of Gravity on the Nervous System. Into Space—A Journey of How Humans Adapt and Live in Microgravity.

[29] Isakovic J., Dobbs-Dixon I., Chaudhury D., Mitrecic D. (2018). Modeling of inhomogeneous electromagnetic fields in the nervous system: A novel paradigm in understanding cell interactions, disease etiology and therapy. Sci. Rep..

[30] Erecińska M., Silver I.A. (1989). ATP and brain function. J. Cereb. Blood Flow Metab. Off. J. Int. Soc. Cereb. Blood Flow Metab..

[31] Attwell D., Laughlin S.B. (2001). An energy budget for signaling in the grey matter of the brain. J. Cereb. Blood Flow Metab. Off. J. Int. Soc. Cereb. Blood Flow Metab..

[32] Raichle M.E., Gusnard D.A. (2002). Appraising the brain’s energy budget. Proc. Natl. Acad. Sci. USA.

[33] Buzsáki G., Anastassiou C.A., Koch C. (2012). The origin of extracellular fields and currents—EEG, ECoG, LFP and spikes. Nature reviews. Neuroscience.

[34] Magistretti P.J., Allaman I. (2015). A cellular perspective on brain energy metabolism and functional imaging. Neuron.

[35] Raichle M.E., Mintun M.A. (2006). Brain work and brain imaging. Annu. Rev. Neurosci..

[36] Wong-Riley M.T. (2010). Energy metabolism of the visual system. Eye Brain.

[37] Rees G. (2007). Neural correlates of the contents of visual awareness in humans. Philos. Trans. R. Soc. Lond. Ser. B Biol. Sci..

[38] Buxton R.B. (2013). The physics of functional magnetic resonance imaging (fMRI). Rep. Prog. Phys. Phys. Soc..

[39] Nunez P.L., Srinivasan R. (2006). Electric Fields of the Brain: The Neurophysics of EEG.

[40] Rees G. (2001). Neuroimaging of visual awareness in patients and normal subjects. Curr. Opin. Neurobiol..

[41] Deco G., Rolls E.T., Romo R. (2009). Stochastic dynamics as a principle of brain function. Prog. Neurobiol..

[42] Herculano-Houzel S. (2012). The remarkable, yet not extraordinary, human brain as a scaled-up primate brain and its associated cost. Proc. Natl. Acad. Sci. USA.

[43] Peng H., Xie P., Liu L., Kuang X., Wang Y., Qu L., Gong H., Jiang S., Li A., Ruan Z. (2021). Morphological diversity of single neurons in molecularly defined cell types. Nature.

[44] Fodstad H. (2001). The neuron theory. Stereotact. Funct. Neurosurg..

[45] Thatcher J.D. (2013). Charged membranes. Sci. Signal..

[46] Harris J.J., Jolivet R., Attwell D. (2012). Synaptic energy use and supply. Neuron.

[47] Zhang X.C., Yang H., Liu Z., Sun F. (2018). Thermodynamics of voltage-gated ion channels. Biophys. Rep..

[48] Raghavan M., Fee D., Barkhaus P.E. (2019). Generation and propagation of the action potential. Handb. Clin. Neurol..

[49] Sibson N.R., Dhankhar A., Mason G.F., Rothman D.L., Behar K.L., Shulman R.G. (1998). Stoichiometric coupling of brain glucose metabolism and glutamatergic neuronal activity. Proc. Natl. Acad. Sci. USA.

[50] Buzsáki G., Kaila K., Raichle M. (2007). Inhibition and brain work. Neuron.

[51] Hirokawa N., Niwa S., Tanaka Y. (2010). Molecular motors in neurons: Transport mechanisms and roles in brain function, development, and disease. Neuron.

[52] Javier-Torrent M., Zimmer-Bensch G., Nguyen L. (2021). Mechanical Forces Orchestrate Brain Development. Trends Neurosci..

[53] Anastassiou C.A., Koch C. (2015). Ephaptic coupling to endogenous electric field activity: Why bother?. Curr. Opin. Neurobiol..

[54] Chiang C.C., Shivacharan R.S., Wei X., Gonzalez-Reyes L.E., Durand D.M. (2019). Slow periodic activity in the longitudinal hippocampal slice can self-propagate non-synaptically by a mechanism consistent with ephaptic coupling. J. Physiol..

[55] Ruffini G., Salvador R., Tadayon E., Sanchez-Todo R., Pascual-Leone A., Santarnecchi E. (2020). Realistic modeling of mesoscopic ephaptic coupling in the human brain. PLoS Comput. Biol..

[56] Scholkmann F. (2015). Two emerging topics regarding long-range physical signaling in neurosystems: Membrane nanotubes and electromagnetic fields. J. Integr. Neurosci..

[57] Qiu C., Shivacharan R.S., Zhang M., Durand D.M. (2015). Can Neural Activity Propagate by Endogenous Electrical Field?. J. Neurosci. Off. J. Soc. Neurosci..

[58] Ucar H., Watanabe S., Noguchi J., Morimoto Y., Iino Y., Yagishita S., Takahashi N., Kasai H. (2021). Mechanical actions of dendritic-spine enlargement on presynaptic exocytosis. Nature.

[59] Piatkevich K.D., Bensussen S., Tseng H.A., Shroff S.N., Lopez-Huerta V.G., Park D., Jung E.E., Shemesh O.A., Straub C., Gritton H.J. (2019). Population imaging of neural activity in awake behaving mice. Nature.

[60] Bohr N. (1932). Light and Life. Atomic Physics and Human Knowledge.

[61] Roncaglia A., Cerisola F., Paz J. (2014). Work Measurement as a Generalized Quantum Measurement. Phys. Rev. Lett..

[62] Ball P. (2011). Physics of life: The dawn of quantum biology. Nature.

[63] Marais A., Adams B., Ringsmuth A.K., Ferretti M., Gruber J.M., Hendrikx R., Schuld M., Smith S.L., Sinayskiy I., Krüger T.P.J. (2018). The future of quantum biology. J. R. Soc. Interface.

[64] Romero E., Prior J., Chin A.W., Morgan S.E., Novoderezhkin V.I., Plenio M.B., van Grondelle R. (2017). Quantum—Coherent dynamics in photosynthetic charge separation revealed by wavelet analysis. Sci. Rep..

[65] Beck F., Eccles J. (1992). Quantum aspects of brain activity and the role of consciousness. Proc. Natl. Acad. Sci. USA.

[66] Tarlacı S., Pregnolato M. (2016). Quantum neurophysics: From non-living matter to quantum neurobiology and psychopathology. Int. J. Psychophysiol. Off. J. Int. Organ. Psychophysiol..

[67] Hameroff S., Penrose R. (2014). Consciousness in the universe: A review of the ‘Orch OR’ theory. Phys. Life Rev..

[68] Fisher M. (2015). Quantum cognition: The possibility of processing with nuclear spins in the brain. Ann. Phys..

[69] Sergi A., Messina A., Vicario C.M., Martino G. (2023). A Quantum-Classical Model of Brain Dynamics. Entropy.

[70] Sahu S., Ghosh S., Fujita D., Bandyopadhyay A. (2014). Live visualizations of single isolated tubulin protein self-assembly via tunneling current: Effect of electromagnetic pumping during spontaneous growth of microtubule. Sci. Rep..

[71] Goodson H.V., Jonasson E.M. (2018). Microtubules and Microtubule-Associated Proteins. Cold Spring Harb. Perspect. Biol..

[72] Kalra A.P., Benny A., Travis S.M., Zizzi E.A., Morales-Sanchez A., Oblinsky D.G., Craddock T.J.A., Hameroff S.R., MacIver M.B., Tuszyński J.A. (2023). Electronic Energy Migration in Microtubules. ACS Cent. Sci..

[73] Roy C., Sherrington C. (1890). On the regulation of the blood supply in the brain. J. Physiol..

[74] Sokoloff L. (1977). Relation between physiological function and energy metabolism in the central nervous system. J. Neurochem..

[75] Poznanski R. (2024). The dynamic organicity theory of consciousness: How consciousness arises from the functionality of multiscale complexity in the material brain. J. Multiscale Neurosci..

[76] Aalling N.N., Nedergaard M., DiNuzzo M. (2018). Cerebral Metabolic Changes During Sleep. Curr. Neurol. Neurosci. Rep..

[77] Shulman R., Hyder F., Rothman D. (2009). Baseline brain energy supports the state of consciousness. Proc. Natl. Acad. Sci. USA.

[78] Dinuzzo M., Nedergaard M. (2017). Brain energetics during the sleep–wake cycle. Curr. Opin. Neurobiol..

[79] Slupe A.M., Kirsch J.R. (2018). Effects of anesthesia on cerebral blood flow, metabolism, and neuroprotection. J. Cereb. Blood Flow Metab. Off. J. Int. Soc. Cereb. Blood Flow Metab..

[80] Stender J., Mortensen K.N., Thibaut A., Darkner S., Laureys S., Gjedde A., Kupers R. (2016). The Minimal Energetic Requirement of Sustained Awareness after Brain Injury. Curr. Biol. CB.

[81] Bazzigaluppi P., Amini A.E., Weisspapier I., Stefanovic B., Carlen P. (2017). Hungry neurons: Metabolic insights on seizure dynamics. Int. J. Mol. Sci..

[82] Pai A., Heining M. (2007). Ketamine. Contin. Educ. Anaesth. Crit. Care Pain.

[83] Juan E., Górska U., Kozma C., Papantonatos C., Bugnon T., Denis C., Kremen V., Worrell G., Struck A.F., Bateman L.M. (2022). Distinct signatures of loss of consciousness in focal impaired awareness versus tonic-clonic seizures. Brain J. Neurol..

[84] Nagel T. (1974). What is it like to be a bat?. Philos. Rev..

[85] Teasdale G., Jennett B. (1974). Assessment of coma and impaired consciousness. A practical scale. Lancet.

[86] Bodart O., Gosseries O., Wannez S., Thibaut A., Annen J., Boly M., Rosanova M., Casali A.G., Casarotto S., Tononi G. (2017). Measures of metabolism and complexity in the brain of patients with disorders of consciousness. NeuroImage. Clin..

[87] Jang H., Mashour G.A., Hudetz A.G., Huang Z. (2024). Measuring the dynamic balance of integration and segregation underlying consciousness, anesthesia, and sleep in humans. Nat. Commun..

[88] Casali A.G., Gosseries O., Rosanova M., Boly M., Sarasso S., Casali K.R., Casarotto S., Bruno M.A., Laureys S., Tononi G. (2013). A theoretically based index of consciousness independent of sensory processing and behavior. Sci. Trans. Med..

[89] Casarotto S., Comanducci A., Rosanova M., Sarasso S. (2016). Stratification of unresponsive patients by an independently validated index of brain complexity. Ann. Neurol..

[90] Mateos D.M., Guevara Erra R., Wennberg R., Perez Velazquez J.L. (2018). Measures of entropy and complexity in altered states of consciousness. Cogn. Neurodyn..

[91] Comolatti R., Pigorini A., Casarotto S., Fecchio M., Faria G., Sarasso S., Rosanova M., Gosseries O., Boly M., Bodart O. (2019). A fast and general method to empirically estimate the complexity of brain responses to transcranial and intracranial stimulations. Brain Stimul..

[92] Tononi G., Edelman G.M. (1998). Consciousness and complexity. Science.

[93] Seth A.K., Izhikevich E., Reeke G.N., Edelman G.M. (2006). Theories and measures of consciousness: An extended framework. Proc. Natl. Acad. Sci. USA.

[94] Sarasso S. (2021). Adenauer Girardi Casali, Silvia Casarotto, Mario Rosanova, Corrado Sinigaglia, Marcello Massimini, Consciousness and complexity: A consilience of evidence. Neurosci. Conscious..

[95] Noel J.P., Simon D., Thelen A., Maier A., Blake R., Wallace M.T. (2018). Probing Electrophysiological Indices of Perceptual Awareness across Unisensory and Multisensory Modalities. J. Cogn. Neurosci..

[96] van Vugt B., Dagnino B., Vartak D., Safaai H., Panzeri S., Dehaene S., Roelfsema P.R. (2018). The threshold for conscious report: Signal loss and response bias in visual and frontal cortex. Science.

[97] Malach R. (2021). Local neuronal relational structures underlying the contents of human conscious experience. Neurosci. Conscious..

[98] Moutard C., Dehaene S., Malach R. (2015). Spontaneous Fluctuations and Non-linear Ignitions: Two Dynamic Faces of Cortical Recurrent Loops. Neuron.

[99] Mashour G.A., Palanca B.J., Basner M., Li D., Wang W., Blain-Moraes S., Lin N., Maier K., Muench M., Tarnal V. (2021). Recovery of consciousness and cognition after general anesthesia in humans. eLife.

[100] Edelman G.M., Gally J.A. (2013). Reentry: A key mechanism for integration of brain function. Front. Integr. Neurosci..

[101] Lamme V.A., Roelfsema P.R. (2000). The distinct modes of vision offered by feedforward and recurrent processing. Trends Neurosci..

[102] Hameroff S.R., Craddock T.J., Tuszynski J.A. (2014). Quantum effects in the understanding of consciousness. J. Integr. Neurosci..

[103] Georgiev D.D., Glazebrook J.F. (2018). The quantum physics of synaptic communication via the SNARE protein complex. Prog. Biophys. Mol. Biol..

[104] Kerskens C., Pérez D. (2022). Experimental indications of non-classical brain functions. J. Phys. Commun..

[105] Chalmers D.J. (2010). Facing Up to the Problem of Consciousness. The Character of Consciousness.

[106] Demertzi A., Tagliazucchi E., Dehaene S., Deco G., Barttfeld P., Raimondo F., Martial C., Fernández-Espejo D., Rohaut B., Voss H.U. (2019). Human consciousness is supported by dynamic complex patterns of brain signal coordination. Sci. Adv..

[107] Kent L., Wittmann M. (2021). Special Issue: Consciousness science and its theories Time consciousness: The missing link in theories of consciousness. Neurosci. Conscious..

[108] Bressler S.L., Kelso J.A. (2016). Coordination Dynamics in Cognitive Neuroscience. Front. Neurosci..

[109] Vatansever D., Schröter M., Adapa R.M., Bullmore E.T., Menon D.K., Stamatakis E.A. (2020). Reorganisation of Brain Hubs across Altered States of Consciousness. Sci. Rep..

[110] Zahedi A., Lynn S.J., Sommer W. (2024). Cognitive simulation along with neural adaptation explain effects of suggestions: A novel theoretical framework. Front. Psychol..

[111] Deco G., Tononi G., Boly M., Kringelbach M.L. (2015). Rethinking segregation and integration: Contributions of whole-brain modelling. Nat. Rev. Neurosci..

[112] Fair D.A., Dosenbach N.U., Church J.A., Cohen A.L., Brahmbhatt S., Miezin F.M., Barch D.M., Raichle M.E., Petersen S.E., Schlaggar B.L. (2007). Development of distinct control networks through segregation and integration. Proc. Natl. Acad. Sci. USA.

[113] Rizkallah J., Annen J., Modolo J., Gosseries O., Benquet P., Mortaheb S., Amoud H., Cassol H., Mheich A., Thibaut A. (2019). Decreased integration of EEG source-space networks in disorders of consciousness. NeuroImage. Clin..

[114] Lord L.D., Stevner A.B., Deco G., Kringelbach M.L. (2017). Understanding principles of integration and segregation using whole-brain computational connectomics: Implications for neuropsychiatric disorders. Philos. Trans. R. Soc..

[115] Wang Y., Hu X., Li Y. (2022). Investigating cognitive flexibility deficit in schizophrenia using task-based whole-brain functional connectivity. Front. Psychiatry.

[116] Koch C., Massimini M., Boly M., Tononi G. (2016). Neural correlates of consciousness: Progress and problems. Nat. Rev. Neurosci..

[117] Tononi G., Sporns O., Edelman G.M. (1994). A measure for brain complexity: Relating functional segregation and integration in the nervous system. Proc. Natl. Acad. Sci. USA.

[118] Del Pozo S.M., Laufs H., Bonhomme V., Laureys S., Balenzuela P., Tagliazucchi E. (2021). Unconsciousness reconfigures modular brain network dynamics. Chaos.

[119] Song C. (2021). Brain structural complexity and consciousness. Philos. Mind Sci..

[120] Cabanac M., Cabanac A.J., Parent A. (2009). The emergence of consciousness in phylogeny. Behav. Brain Res..

[121] Mason G.J., Lavery J.M. (2022). What Is It Like to Be a Bass? Red Herrings, Fish Pain and the Study of Animal Sentience. Front. Vet. Sci..

[122] Lewin R. (1980). Is your brain really necessary?. Science.

[123] Ferris C.F., Cai X., Qiao J., Switzer B., Baun J., Morrison T., Iriah S., Madularu D., Sinkevicius K.W., Kulkarni P. (2019). Life without a brain: Neuroradiological and behavioral evidence of neuroplasticity necessary to sustain brain function in the face of severe hydrocephalus. Sci. Rep..

[124] Sprengers M., Vonck K., Carrette E., Marson A.G., Boon P. (2017). Deep brain and cortical stimulation for epilepsy. Cochrane Database Syst. Rev..

[125] Burke M.J., Fried P.J., Pascual-Leone A. (2019). Transcranial magnetic stimulation: Neurophysiological and clinical applications. Handb. Clin. Neurol..

[126] Berglund K., Stern M.A., Gross R.E. (2021). Bioluminescence-Optogenetics. Adv. Exp. Med. Biol..

[127] Vakani R., Nair D.R. (2019). Electrocorticography and functional mapping. Handb. Clin. Neurol..

[128] Müller-Putz G.R. (2020). Electroencephalography. Handb. Clin. Neurol..

[129] Wang Y., Wang R., Xu X. (2017). Neural Energy Supply-Consumption Properties Based on Hodgkin-Huxley Model. Neural Plast.

[130] Nath S. (2021). Energy landscapes and dynamics of ion translocation through membrane transporters: A meeting ground for physics, chemistry, and biology. J. Biol. Phys..

[131] Zhu F., Wang R., Pan X., Zhu Z. (2019). Energy expenditure computation of a single bursting neuron. Cogn. Neurodyn..

[132] Liput D.J., Nguyen T.A., Augustin S.M., Lee J.O., Vogel S.S. (2020). A Guide to Fluorescence. Lifetime Microscopy and Förster’s Resonance Energy Transfer in Neuroscience. Curr. Protoc. Neurosci..

[133] Barrett A.B., Seth A.K. (2011). Practical measures of integrated information for time-series data. PLoS Comput. Biol..

[134] Sanz Perl Y., Bocaccio H., Pallavicini C., Pérez-Ipiña I., Laureys S., Laufs H., Kringelbach M., Deco G., Tagliazucchi E. (2021). Nonequilibrium brain dynamics as a signature of consciousness. Phys. Rev. E.

[135] Wutzl B., Golaszewski S.M., Leibnitz K., Langthaler P.B., Kunz A.B., Leis S., Schwenker K., Thomschewski A., Bergmann J., Trinka E. (2021). Narrative Review: Quantitative EEG in Disorders of Consciousness. Brain Sci..

[136] Bassett D.S., Sporns O. (2017). Network neuroscience. Nat. Neurosci..

[137] Bartlett G. (2022). Does integrated information theory make testable predictions about the role of silent neurons in consciousness?. Neurosci. Conscious..

[138] Pockett S. (2012). The electromagnetic field theory of consciousness: A testable hypothesis about the characteristics of conscious as opposed to non-conscious fields. J. Conscious. Stud..

[139] McFadden J. (2020). Integrating information in the brain’s EM field: The cemi field theory of consciousness. Neurosci. Conscious..

[140] Hunt T., Schooler J.W. (2019). The Easy Part of the Hard Problem: A Resonance Theory of Consciousness. Front. Hum. Neurosci..

[141] Solms M. (2019). The Hard Problem of Consciousness and the Free Energy Principle. Front. Psychol..

[142] Friston K. (2010). The free-energy principle: A unified brain theory?. Nat. Rev. Neurosci..

[143] Friston K.J., Wiese W., Hobson J.A. (2020). Sentience and the Origins of Consciousness: From Cartesian Duality to Markovian Monism. Entropy.

[144] Pepperell R. (2018). Consciousness as a physical process caused by the organisation of energy in the brain. Front. Psychol..

